# SEER and Gene Expression Data Analysis Deciphers Racial Disparity Patterns in Prostate Cancer Mortality and the Public Health Implication

**DOI:** 10.1038/s41598-020-63764-4

**Published:** 2020-04-22

**Authors:** Wensheng Zhang, Yan Dong, Oliver Sartor, Erik K. Flemington, Kun Zhang

**Affiliations:** 1 Bioinformatics Core of Xavier NIH RCMI Center of Cancer Research; Department of Computer Science, Xavier University of Louisiana, New Orleans, 70125 LA USA; 2 Department of Structural and Cellular Biology, Tulane University School of Medicine, Tulane Cancer Center, New Orleans, 70112 LA USA; 3 Department of Medicine, Tulane University School of Medicine, Tulane Cancer Center, New Orleans, 70112 LA USA; 4 Department of Pathology, Tulane University School of Medicine, Tulane Cancer Center, New Orleans, 70112 LA USA

**Keywords:** Cancer epidemiology, Prostate cancer, Data mining, Prostate

## Abstract

A major racial disparity in prostate cancer (PCa) is that African American (AA) patients have a higher mortality rate than European American (EA) patients. We filtered the SEER 2009–2011 records and divided them into four groups regarding patient races and cancer grades. On such a partition, we performed a series of statistical analyses to further clarify the aforementioned disparity. Molecular evidence for a primary result of the epidemiological analysis was obtained from gene expression data. The results include: (1) Based on the registry-specific measures, a significant linear regression of total mortality rate (as well as PCa specific mortality rate) on the percentage of (Gleason pattern-based) high-grade cancers (PHG) is demonstrated in EAs (p < 0.01) but not in AAs; (2) PHG and its racial disparity are differentiated across ages and the groups defined by patient outcomes; (3) For patients with cancers in the same grade category, i.e. the high or low grade, the survival stratification between races is not significant in most geographical areas; and (4) The genes differentially expressed between AAs’ and EAs’ tumors of the same grade category are relatively rare. The perception that prostate tumors are more lethal in AAs than in EAs is reasonable regarding AAs’ higher PHG, while high grade alone could not imply aggressiveness. However, this perception is questionable when the comparison is focused on cases within the same grade category. Supporting observations for this conclusion hold a remarkable implication for erasing racial disparity in PCa. That is, “Equal grade, equal outcomes” is not only a verifiable hypothesis but also an achievable public health goal.

## Introduction

Prostate cancer (PCa) is the most commonly diagnosed non-skin cancer and the second leading cause of cancer mortality in American men^1^. Adenocarcinomas amount to 95% of PCa cases^2^. Racial disparities in this cancer type have been revealed by numerous epidemiological studies^3–5^. One of those disparities is that African American (AA) patients have a higher mortality rate than their European American (EA) counterparts. A popular explanation for this disparity is that prostate tumors may be more aggressive in AAs than in EAs^6–8^. Molecular evidence for such an explanation has been reported by several studies in the past years^9–11^.

Nevertheless, the validity of the notion that prostate tumors may be more aggressive in AAs than in EAs is severely compromised by the “Equal care, equal outcomes” phenomenon or conception, as demonstrated in^12–14^. For example, Fowler and Terrell (12) reviewed the outcomes of 148 black and 209 white men with localized prostate cancer treated with surgery or radiation therapy over an 11-year period at a Veterans Affairs medical center. Their results showed that, after a median follow-up of 96 months, there were no significant differences in all-cause, cause specific, metastasis-free, clinical disease-free, or prostate-specific antigen recurrence-free survival rates in 109 black and 167 white men with low-stage cancer treated with surgery or radiation therapy, or in 39 black and 42 white men with high-stage cancer treated with radiotherapy.

On the other hand, the conception of “Equal care, equal outcomes” has its own problem. That is, it is often a conclusion drawn from the results of limited-size cohorts. To achieve a more comprehensive and accurate understanding of the racial disparities in the outcomes of PCa patients, we performed an integrative analysis of large-scale clinical data collected by the Surveillance, Epidemiology, and End Results Program (SEER) and the gene expression data deposited in the Cancer Genome Atlas (TCGA) and Gene Expression Omnibus (GEO) databases. Most importantly, in addition to other research objectives, we primarily aim to test the “Equal grade, equal outcomes” hypothesis, namely that no disparity in mortality exists for patients whose cancer is in the same grade category (low or high).

## Materials and Methods

### Study design

In this study, we used three sources of data, i.e., the SEER data, the TCGA digital gene expression data (Ex-1), and an aggregation of three GEO microarray gene expression datasets (Ex-2). The samples in all of these datasets compose patients of multiple races, facilitating disparity research, especially the test of the proposed hypothesis. Our statistical analysis focused on the samples from non-Latin European American (EA) and African Americans (AA) populations. The integration of the SEER data with the gene expression data was achieved by an approximate alignment of the cancer grades via Gleason patterns (GP). That is, the high-grade or low-grade cancers in the SEER data correspond to the GP-4 or GP-3 specimens in the gene expression data. Specifically, we partitioned the SEER samples into four groups, i.e., AA-LG, AA-HG, EA-LG and EA-HG, where HG and LG denote the high-grade and low-grade cancer (patient), respectively. Our primary results or conclusions for a specific research question were derived by performing pairwise comparisons of those four groups. The gene expression data were further analyzed to test if a biological basis, inferred from the stratification of gene expression levels across populations and Gleason patterns, exists for the SEER data-based results about mortality disparity patterns.

### SEER data

We retrieved prostate cancer SEER data from the 2009–2011 database, and then refined the data to generate a working dataset (SEER-WD), containing 86996 AA and non-Hispanic EA cancer cases collected by 48 registries (including 59 patients whose survival or follow-up times are unknown). Each registry represents a county or a parish in California, Louisiana and other five states. These registries are selected as each of them has at least 100 AA or EA patients documented during the studied time period (Table 1). Because the patients in the selected data entered into the survey during a short time span (i.e. three years), we can assume that the individuals of a specific race in each registry constitute a cohort whose socio-economic relevance is relatively strong. Moreover, the potential influence of the Hurricane Katrina occurring in Louisiana in 2005 could be largely alleviated regarding the studied period.

**Table 1:** SEER prostate cancer incidence and death statistics in 48 registries during 2009–2011^‡^.

Registry ID	State	County	Incidence Number				Death Number^§^			
			AA-LG	AA-HG	EA-LG	EA-HG	AA-LG	AA-HG	EA-LG	EA-HG
6001	CA	Alameda	235	297	736	833	13 (0)	58 (23)	53 (8)	116 (47)
6013	CA	Contra Costa	106	149	737	897	9 (0)	30 (11)	50 (1)	116 (46)
6019	CA	Fresno	42	58	486	558	9 (1)	14 (7)	41 (2)	87 (32)
6037	CA	Los Angeles	933	1430	4511	5583	83 (9)	236 (88)	328 (45)	770 (308)
6059	CA	Orange	52	85	1665	2471	6 (1)	9 (5)	127 (11)	298 (118)
6065	CA	Riverside	155	148	1298	1528	15 (4)	25 (10)	120 (18)	219 (97)
6067	CA	Sacramento	133	228	817	984	12 (1)	23 (11)	57 (9)	145 (54)
6071	CA	San Bernardino	167	235	1159	1189	11 (3)	48 (18)	118 (14)	208 (86)
6073	CA	San Diego	140	250	1537	2565	13 (2)	36 (13)	134 (6)	404 (150)
6075	CA	San Francisco	74	82	401	393	3 (0)	16 (12)	33 (5)	52 (23)
6077	CA	San Joaquin	37	67	390	503	6 (1)	8 (3)	54 (2)	74 (21)
6085	CA	Santa Clara	59	79	1265	1352	3 (0)	6 (4)	68 (6)	184 (60)
6095	CA	Solano	84	122	248	298	5 (0)	17 (3)	29 (2)	40 (14)
9001	CT	Fairfield	98	127	845	971	10 (1)	17 (9)	46 (0)	116 (37)
9003	CT	Hartford	144	173	657	901	10 (0)	21 (8)	63 (5)	133 (51)
9009	CT	New Haven	102	119	579	933	9 (0)	16 (5)	38 (6)	141 (53)
13021	GA	Bibb	84	124	85	112	6 (2)	20 (3)	10 (1)	23 (5)
13051	GA	Chatham	100	122	127	153	9 (1)	21 (4)	9 (0)	23 (6)
13063	GA	Clayton	101	169	63	65	7 (0)	25 (14)	4 (0)	12 (3)
13067	GA	Cobb	94	176	436	512	3 (0)	20 (11)	26 (0)	59 (24)
13089	GA	DeKalb	272	554	225	291	11 (2)	85 (33)	11 (0)	49 (13)
13095	GA	Dougherty	83	81	56	60	11 (1)	13 (6)	3 (1)	16 (3)
13121	GA	Fulton	369	671	439	472	46 (8)	136 (57)	18 (2)	55 (14)
13135	GA	Gwinnett	83	166	357	474	1 (0)	17 (7)	16 (0)	51 (16)
13151	GA	Henry	76	88	123	117	3 (0)	9 (2)	7 (0)	17 (1)
13215	GA	Muscogee	97	171	75	124	12 (1)	28 (8)	9 (3)	27 (8)
13245	GA	Richmond	67	161	50	84	8 (2)	22 (9)	4 (0)	15 (9)
22017	LA	Caddo	77	227	107	262	11 (0)	40 (15)	7 (0)	47 (12)
22019	LA	Calcasieu	53	75	176	157	6 (0)	14 (2)	15 (0)	23 (7)
22033	LA	East Baton Rouge	268	207	372	327	36 (3)	35 (9)	29 (2)	39 (9)
22051	LA	Jefferson	121	178	292	417	12 (0)	30 (6)	28 (3)	62 (19)
22071	LA	Orleans	245	315	102	144	24 (3)	51 (27)	8 (0)	19 (8)
26099	MI	Macomb	48	83	658	1316	4 (0)	16 (6)	57 (2)	190 (55)
26125	MI	Oakland	178	385	1133	1761	10 (0)	49 (14)	68 (3)	219 (57)
26163	MI	Wayne	801	1531	1027	1617	87 (7)	305 (88)	84 (2)	260 (71)
34001	NJ	Atlantic	57	62	268	241	5 (0)	12 (4)	21 (1)	36 (13)
34003	NJ	Bergen	72	75	1009	754	5 (0)	7 (4)	51 (9)	100 (33)
34005	NJ	Burlington	116	157	470	510	12 (0)	17 (7)	34 (0)	83 (27)
34007	NJ	Camden	97	180	405	511	5 (1)	28 (13)	23 (1)	64 (27)
34013	NJ	Essex	385	382	504	444	39 (3)	59 (26)	31 (2)	51 (21)
34017	NJ	Hudson	79	86	303	308	6 (1)	22 (12)	35 (4)	62 (27)
34021	NJ	Mercer	124	118	305	322	14 (0)	19 (8)	18 (1)	43 (12)
34023	NJ	Middlesex	82	115	595	610	7 (1)	15 (5)	47 (5)	101 (30)
34025	NJ	Monmouth	55	78	787	730	2 (0)	15 (6)	43 (2)	99 (18)
34031	NJ	Passaic	56	67	446	374	7 (2)	12 (3)	37 (2)	49 (20)
34039	NJ	Union	140	185	448	413	12 (3)	34 (12)	30 (3)	51 (9)
53033	WA	King	81	193	1241	1716	5 (0)	21 (9)	66 (6)	214 (78)
53053	WA	Pierce	56	94	563	958	6 (0)	20 (7)	32 (2)	129 (45)

^‡^AA-LG and AA-HG: African American High and Low Grade. EA-LG and EA-HG: European American High and Low Grade. ^§^In parenthesis are the numbers of prostate cancer specific deaths.

The SEER determines the *stage* of a PCa patient according to the histologic grade of his disease tissue. In the grading system, the codes I, II, III and IV denote “well differentiated”, “moderately differentiated”, “poorly differentiated” and “undifferentiated; anaplastic”, respectively. The cases in these four grades respectively account for 1.1%, 42.3%, 56.3% and 2.8% of the total records in SEER-WD. About 88% of the cases have the survival or follow-up times falling into the interval of 36 and 72 months (Supplementary Fig. S1). According to the SEER Program Coding and Staging Manual 2012^15^, the cancers coded with I, II and III have Gleason Scores (GS) ranking from 2 to 4, 5 to 6 and 7 to 10, respectively (GS corresponding to the code IV is missed in the Manual but it should be over 8). In this study, we combined I and II into the low-grade category (LG), and combined III and IV into the high-grade category (HG). The main difference between a HG patient and a LG patient is that a representative tumor specimen from the former but not from the latter contains Gleason patterns 4 or 5 (GP-4 or -5) as the primary or second prevalent ones. It is well known that tumorous cells in GP-4 and GP-5 are more aggressive than those in GP-3^16–18^. Therefore, HG and LG could be considered as two prostate cancer “subtypes” with inequivalent lethality.

### Calculation of mortality metrics

Total mortality rate (TMR) and prostate cancer specific mortality rate (PSMR) are calculated using the formula TMR = M/T and PSMR = M1/T, respectively. Here, T is the total number of the patients; M is the number of patients with the values in the “Vital status recode (study cutoff used)” column of the SEER data being “Dead”; and M1 is the number of patients with the values in the “SEER cause-specific death classification” column of the SEER data being “Dead (attributable to this cancer dx)”.

### Digital gene expression data (Ex-1)

Ex-1 contains the log2 transformed level-3 digital (RNA-seq) gene expression profiling of 333 TCGA prostate adenocarcinomas samples whose Gleason patterns (GPs) were reviewed/corrected by two pathologists^19^. A refined version of this dataset, which includes 65 GS-6(3 + 3), 102 GS-7(3 + 4), 78 GS-7(4 + 3) and 44 GS-8(4 + 4) tumors, is focused on in our study. TCGA quantified and normalized the gene expression levels using the RSEM (RNASeq by Expectation Maximization) method^20,21^.

### Microarray gene expression data (Ex-2)

Ex-2 is a composite dataset containing the clinical and gene expression information of primary prostate cancers in three cohorts: a section of GSE21034^22^, GSE62667^23^, and GSE72291^24^. These three cohorts are respectively consisted of 131, 182 and 139 samples. Together, the numbers of GS-5, -6, -7, -8, -9, and -10 tumors are 1, 86, 265, 54, 42 and 2, respectively. There are two tumors whose GSs are missing in the clinical data. In order to reduce unnecessary complexities in statistical analysis and succinctly present the results, we further ignored the difference between GS-5 and GS-6 tumors (i.e. treat both of them as “GS-6”) and the difference among GS-8, GS-9 and GS-10 ones (i.e. treat all of them as “GS-8”). The gene expression levels of all these tumors were measured by the same platform, i.e. Affymetrix Human Exon 1.0 ST Arrays. We first downloaded the raw data from the GEO database and then used the frozen Robust Multi-array Analysis algorithm^25^ to summarize and normalize the transcriptomic quantities to generate gene-level expression profiling of each individual sample. Finally, the quantile normalization was repeatedly applied to the expression matrix of the 452 samples.

### Data augmentation

While Ex-1 and Ex-2 are the largest datasets available to us for studying racial disparities in gene expression of PCa tissues, our research is still subject to a challenge arising from data limitations. That is, the numbers of samples from the interested minority population, i.e. AA, is not sufficiently large. In both datasets, only 12–13% of tumor samples are from AA patients. This situation is further complicated as these tumors are distributed among four to five Gleason Grade-based categories. In particular, it is inappropriate to use the gene expression information of GS-7 tumors, which account for approximately a half of the samples, in a disparity analysis. The reason is that, for a GS-7 tumor, the Gleason pattern (GP-3 or GP-4) of the specimen used in the RNA-seq or microarray experiment is uncertain. This is different from a GS-6 or GS-8 tumor whose experimental specimen can be heuristically assigned to GP-3 or GP-4 category, respectively. On the other hand, we have identified a strong expression signature of 288 genes for distinguishing GP-4 specimens from GP-3 specimens in another work^26^. As a result, before performing the advanced analysis, we used a machine learning method (see the Statistical Method subsection) to partition the GS-7 tumor samples into the GP-3 and GP-4 specimens. In this way, we substantially augmented the clinical information of the gene expression datasets, which facilitates the comparison of gene expression programs between high-grade and low-grade cancers.

### Statistical methods

We used linear regression to model the relationship between the proportion of high-grade cancers and mortality rate. The differences of survival time between two patient groups were evaluated by Cox-PH regression. T-test was used to identify the differentially expressed genes between two sample categories. The hierarchical clustering analysis was performed using Ward’s method and the Manhattan distance. Fisher’s exact test was adopted to test the independence of two different sample partitions and to evaluate the functional enrichment of significant genes. The employed software includes the relevant functions in R packages “stats”, “gplot” and “survival”, the David tool^27^, and an on-line available R function heatmap.3() (http://www.biostars.org/p/18211/).

We used the Support Vector Machine (SVM) (9) to predict the actual Gleason pattern (GP-3 or GP-4) of the specimen from a GS-7 tumor. A SVM model was trained on a dataset consisting of GS-6 and GS-8 samples. The *svm()* function in the R package “e1071” was implemented with the default parameters except for the class weights and kernel type. We specified the class weights as the reciprocals of the fractions of the GP-4 and GP-3 samples in the training set. The pattern of each GS-7 tumor was predicted twice using the linear and sigmoid kernels, respectively. Only the tumors with consistent predictions were kept for further analysis. Our preliminary study showed that such a double-kernel prediction and filtering can warrant the sensitivity and specificity being over 0.9.

All the data used in this study have been previously published or can be freely obtained from public resources. All analyses were performed using standard statistical methods in accordance with relevant guidelines and regulations. All protocols of experiments and information collection were approved by the National Institute of Health (NIH) when the original owners of the data carried out their studies. The gene expression data meet the minimum information standards. The informed consent was obtained from all prostate cancer patients, who were over 18.

## Results

### Disparity in the regression of TMR on PHG

**Figure 1:**
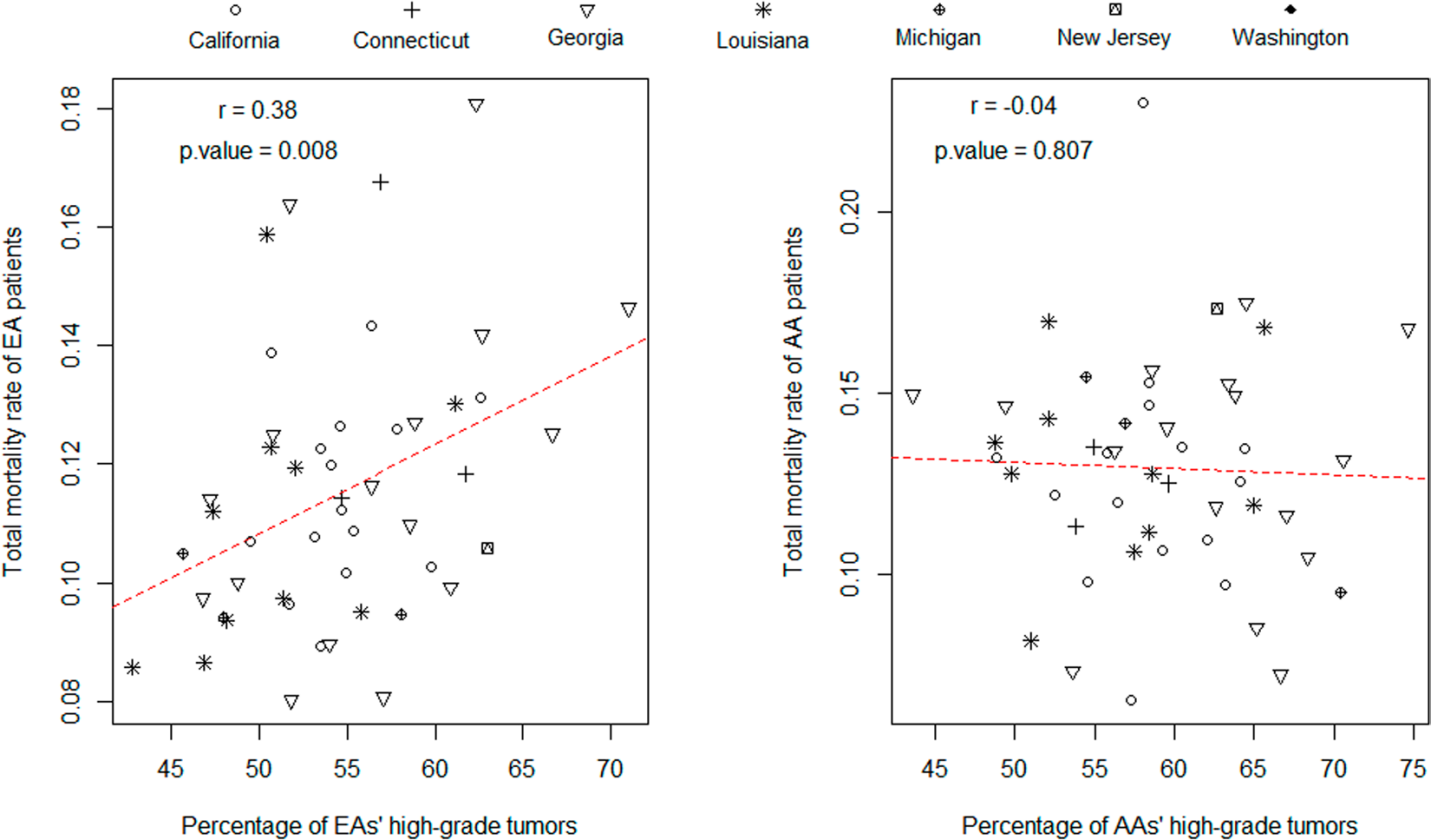
Scatter plots and correlation analysis for registry-specific TMR and PHG measures in the EA (left) and AA (right) populations. The x-axis is PHG and the y-axis is TMR. Each data point represents a registry (i.e. county or parish).

Our results show that the nation-wide TMR and PHG for the EA population are 0.126 and 55.9%, respectively. The corresponding quantities for the AA population are 0.157 and 60.4%, respectively. Registry-specific TMR and PHG, called R-TMR and R-PHG hereafter, are calculated with respect to each individual registry as the “experimental” unit. For EAs, the R-TMRs range from 0.08 to 0.181 and R-PHGs range from 42.7% to 71.0%. For AAs, the R-TMRs range from 0.065 to 0.23 and R-PHGs range from 43.6% to 74.7%. According to a paired t-test, the difference between AAs and EAs is extremely significant in both R-TMR (p = 0.003) and R-PHG (p < 0.000001). As expected, a significant linear regression of R-TMR on R-PHG is observed in EAs (p < 0.01) and the Pearson correlation (r) between these two metrics is up to 0.38. However, such a relationship is not observed in AAs (Fig. 1).

### Disparity in the regression of PSMR on PHG

**Figure 2:**
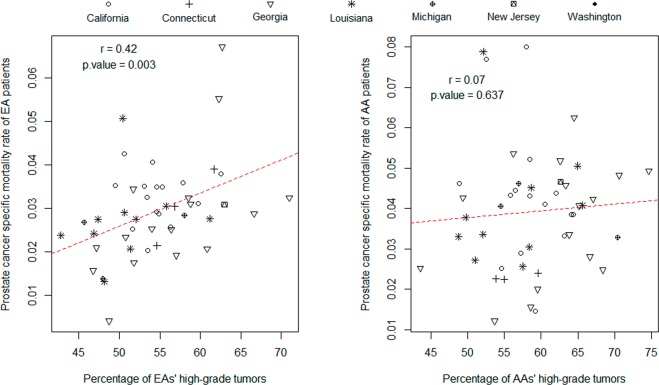
Scatter plots and correlation analysis for registry-specific PSMR and PHG measures in the EA (left) and AA (right) populations. The x-axis is PHG and the y-axis is PSMR. Each data point represents a registry (i.e. county or parish).

Our results show that the nation-wide PSMRs are 0.03 and 0.04 for EA and AA populations, respectively. Registry-specific PSMRs (R-PSMRs) for EAs and AAs range from 0.004 to 0.067 and 0.012 to 0.08, respectively. According to a paired t-test, the difference between AAs and EAs is significant (p < 0.01). Similar to the situation for R-TMR, a significant linear regression of R-PSMR on R-PHG is observed in EAs (r = 0.42, p < 0.003) but not in AAs (Fig. 2).

### Patient age related disparity in PHG

**Figure 3:**
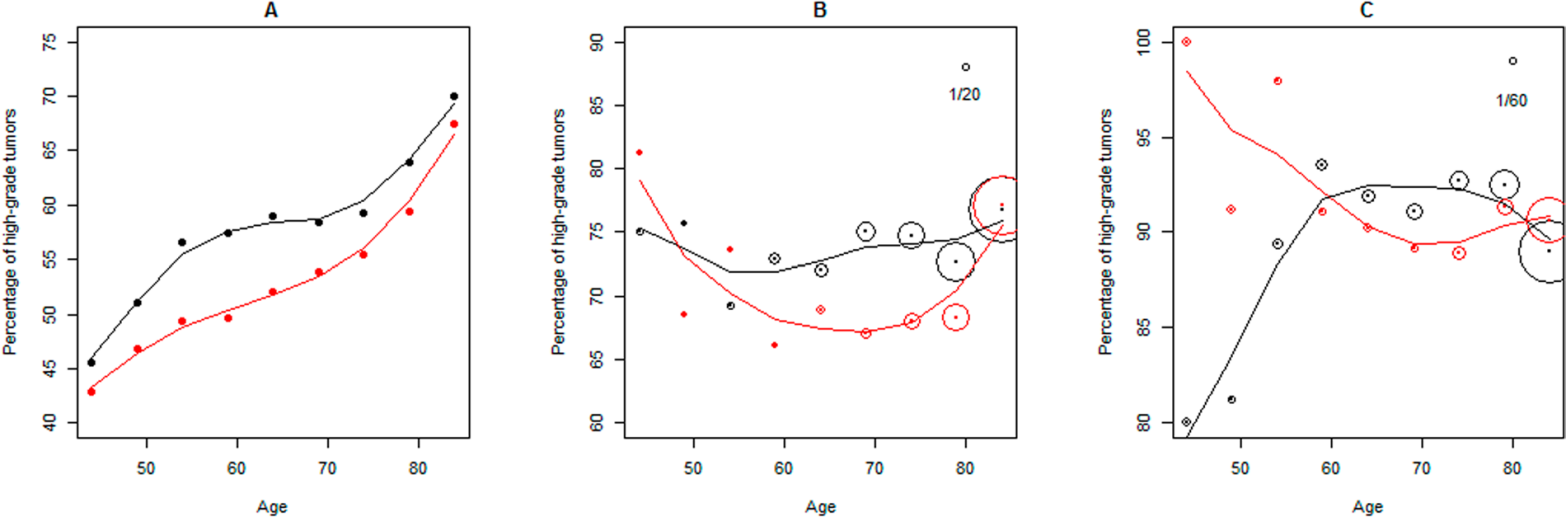
Patient age-related disparity patterns in PHG and cancer mortality metrics. The domain of patient ages at the initial diagnosis is divided into ten segments (i.e. <45, 45–49, 50–54, 55–59,60–64, 65–69, 70–74, 75–79, 80–84 and > =85 years). Race-specific PHG and mortality metrics are calculated for individual age segments. Red and black points/lines represent the EA and AA populations, respectively. In plot A, the information of all patients is used in calculating PHG. In plot B, PHG is calculated with the information of all the patients who died during the follow-up periods; and the diameter of the circle at a data point is proportional to the corresponding TMR (total mortality rate of patients) with the reference being printed at the top right corner. In plot C, PHG is calculated with the information of the patients who died of prostate cancers during the follow-up periods; and the diameter of the circle at a data point is proportional to the corresponding PSMR (prostate cancer specific mortality rate) with the reference being printed at the top right corner.

The domain of patient ages at the initial diagnosis dates are partitioned into ten age segments (A-S), i.e. <45, 45–49, 50–54, 55–59, 60–64, 65–69, 70–74, 75–79, 80–84, and > = 85 years (See Supplementary Fig. 2A, B for the distributions). Race and A-S specific PHGs are calculated and are depicted by a scatter plot. A smooth spline method is used to generate two curves for AA and EA populations, respectively. As shown in Fig. 3A, the area between these two curves assembles a “dolphin” shape with AA curve at the top, EA curve at the bottom, <45 A-S points at the head end and > = 85 A-S points at the tail end. This indicates that PHG is consistently higher in AAs than in EAs but the differences are subtle for the early-onset patients and are almost ignorable for the later-onset patients.

### Patient age and tumor grade-related disparity in cancer mortality

Two subsets are extracted from SEER-WD. The first (WD-1) contains 10077 patients who died during the follow-up periods regardless of the cause of death. The second (WD-2) contains 2825 patients whose deaths were attributable to prostate cancer. Using the same method employed in the previous section, we graphically depict the association patterns between PHG and patient age. The patterns obtained from WD-1 and WD-2 are demonstrated in Fig. 3B, C, respectively. In particular, open circles whose diameters are proportional to the race and A-S specific quantities of two mortality metrics (i.e. TMR and PSMR) are added to these two plots, respectively. The graphics provide us with the following epidemiological implications. First, in the EA population, the patients in the middle age segments (spanning 50–74 years) have a lower PHG than those in the end age segments (<50 or > =75 years), regardless of the death causes. But this is not the case for the AA population, in which the PHG quantities of the patients who died of prostate cancer are almost consistent across the entire age span except for a very low value being observed at the < =45 A-S. Second, disparity regarding the PSMR metric is substantial and consistent over patient ages. Third, as expected, the quantities of these two mortality metrics increase with patient ages. The death cases are mainly observed in the patients whose ages at the initial diagnosis are over 70 years.

### Demographic disparity patterns in grade-related survival stratification

**Figure 4:**
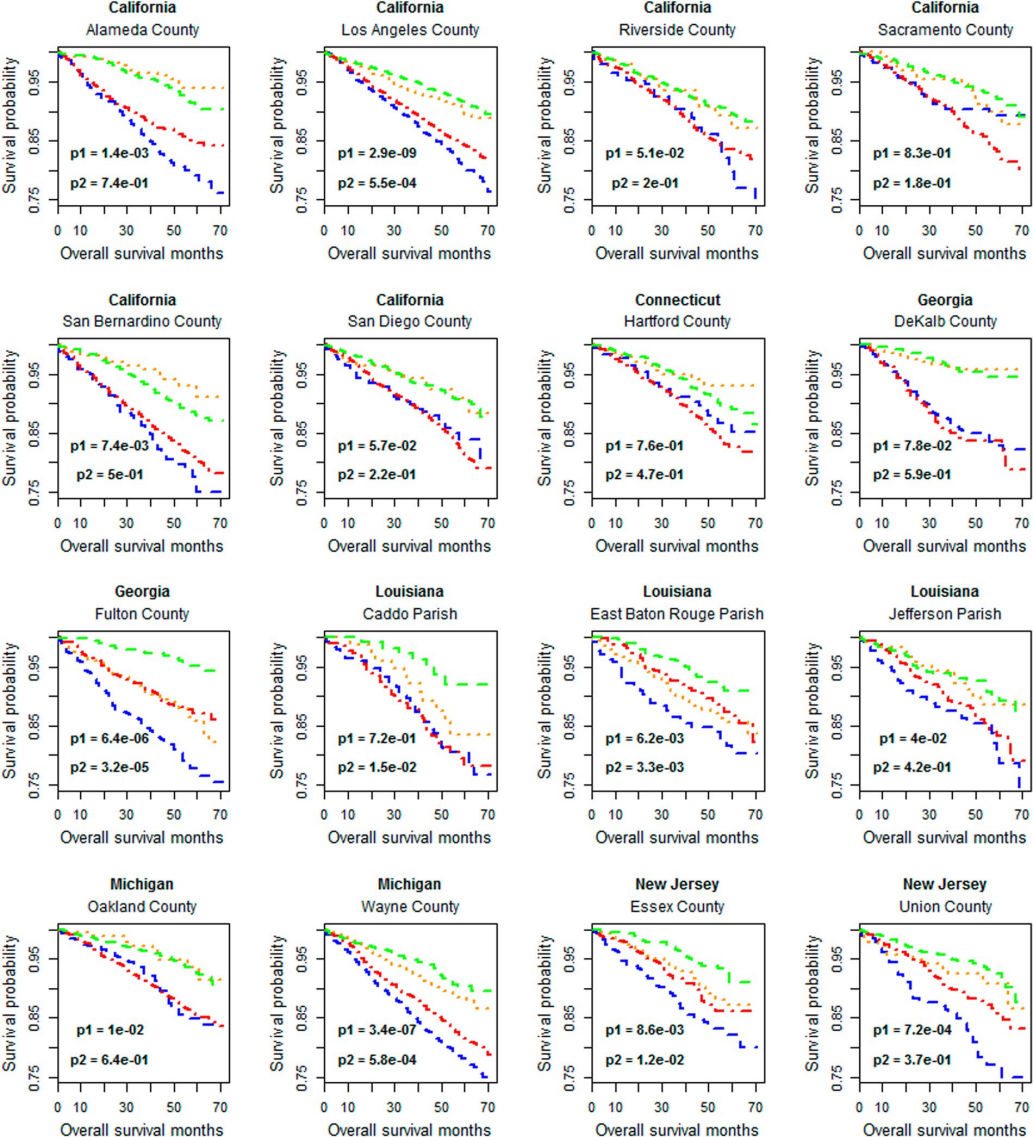
Survival analyses of the patients in 16 representative registries (i.e. counties or parishes) regarding all-causes mortality. Green: EA-LG, i.e. EA patients with low-grade cancers. Red: EA-HG, i.e. EA patients with high-grade cancers. Orange: AA-LG, i.e. AA patients with low-grade cancers. Blue: AA-HG, i.e. AA patients with high-grade cancers. p1: p-value for the comparison between EA-HG and AA-HG. p2: p-value for the comparison between EA-LG and AA-LG.

Among the registries included in SEER-WD, sixteen have at least 280 patients in both EA and AA populations. Geographical area-specific survival analysis is performed on the data of these registries. The statistical significance of the survival stratification of the AA-HG and EA-HG groups, as well as the stratification in AA-LG and EA-LG groups, is evaluated. The results for all-causes mortality are shown in Fig. 4, in which the individual plot displays the Kaplan Meier curves of the patients in a registry. The p-values obtained from Cox-PH regression analysis, in which the age of a patient at the initial diagnosis is included as a covariate, are also printed in the plots. With the criterion of p-value <0.05, the results can be divided into four classes, denoted by **I, II, III** and **IV**, respectively. **I:** Racial survival disparity is observed in patients of both HG and LG (cancer) groups, which is the pattern present in Georgia::Fulton County, Louisiana::East Baton Rouge Parish and other three registries. **II:** The disparity is observed only in patients of HG group, which is the pattern present in California::Alameda County, New Jersey::Essex County and other three registries. **III:** The disparity is observed only in patients of LG group, which is the pattern in Louisiana::Caddo Parish**. IV:** The disparity is not observed in patients of both groups, which is the pattern present in California::Riverside County, Georgia::DeKalb County and other two registries.

The results of survival analysis for prostate cancer specific mortality are shown in Supplementary Fig. S3, in which the patients who died from other causes or whose dead cause categories could not be determined from the data are considered as “censored” cases. The registry-specific survival stratification patterns in Supplementary Fig. S3 are largely consistent with those in Fig. 4, but significant racial disparities (p1 or p2 < 0.05) are only found in 4 (out of 16) registries.

### Contributions of grade and race factors to gene expression variability

This set of analyses is separately performed on the TCGA digital gene expression dataset (Ex-1) and the composited microarray gene expression dataset (Ex-2). The objectives include: (1) evaluating the racial effects on individual gene expression levels of the tumor specimens with the same Gleason pattern (or grade category); and (2) evaluating the effects of Gleason patterns on individual gene expression levels of the tumor specimens of the patients of the same race. Based on the patient races (i.e. AA and EA) and the Gleason patterns (i.e. GP-3 and GP-4) of tumor specimens, we establish four sample groups, i.e. AA&GP-3, AA&GP-4, EA&GP-3 and EA&GP-4. In Ex-1, the sizes of the four specimen groups are 21, 17, 106 and 136, respectively. In Ex-2, the corresponding numbers are 30, 19, 159 and 164, respectively. For the objective (1), we conducted two comparisons, i.e. AA&GP-3 versus EA&GP-3 and AA&GP-4 versus EA&GP-4. For the objective (2), we performed another two comparisons, i.e. AA&GP-3 versus AA&GP-4 and EA&GP-3 versus EA&GP-4.

**Figure 5:**
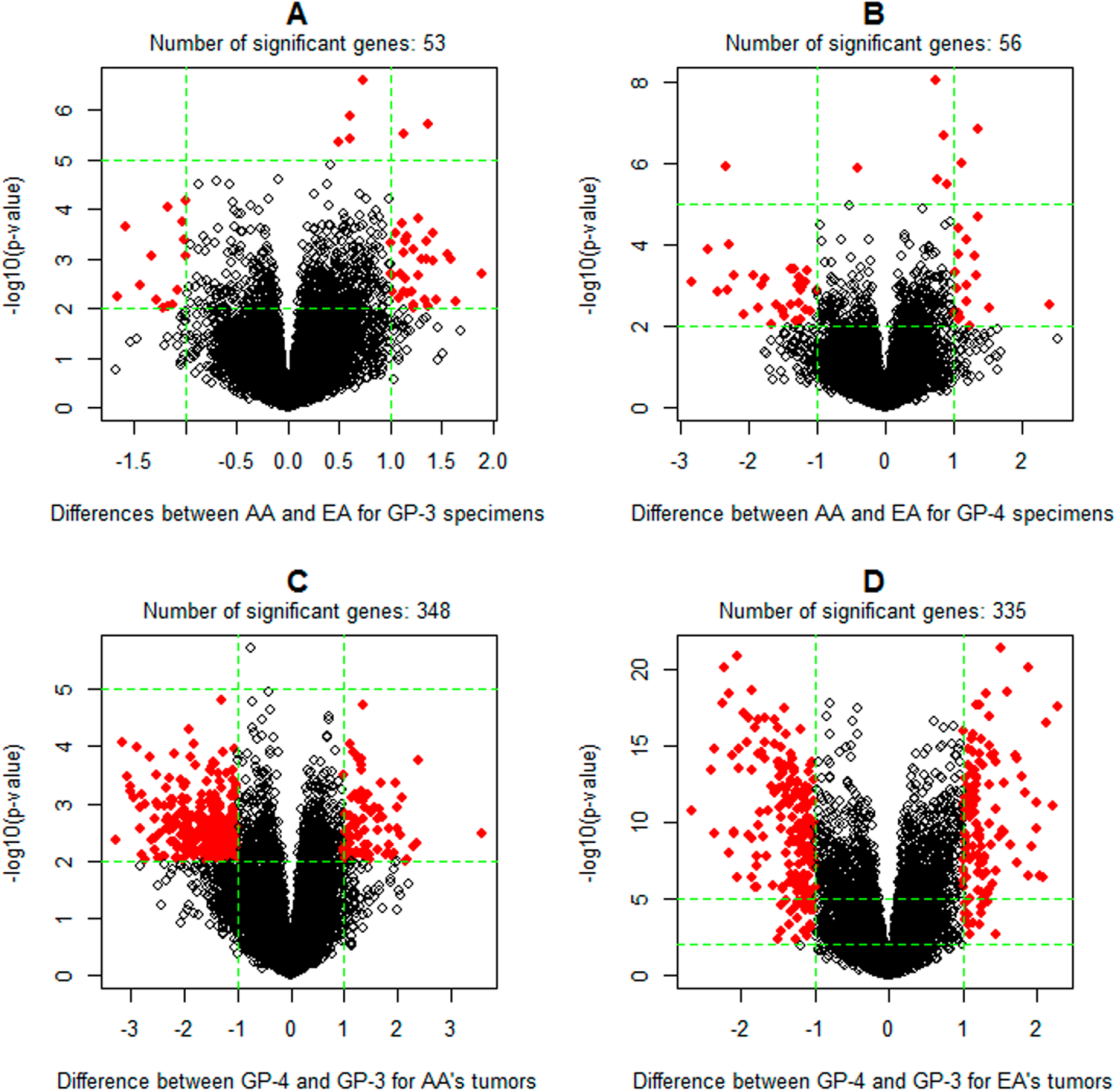
Identification of differentially expressed genes between specimen groups defined by patient races and Gleason patterns. The data set Ex-1, i.e. the TCGA digital expression dataset, is used. Significant genes are indicated by red points. The analysis is based on the log2 transformed gene expression levels. Thus, the cutoffs (1 and -1) correspond to a 2-fold change.

The results obtained from the analysis of dataset Ex-1 are shown in Fig. 5A–D. With the cutoffs of fold change being larger than 2 and p-values being less than 0.01, only 49 and 51 significant genes are identified in AA&GP-3 versus EA&GP-3 and AA&GP-4 versus EA&GP-4, respectively. No KEGG pathway is over-represented by those genes. The lists (i.e. L3 and L4) of significant genes for AA&GP-3 versus AA&GP-4 and EA&GP-3 versus EA&GP-4 are much longer, containing 348 and 335 genes, respectively. Three KEGG pathways are over-represented (p < 0.05, Fold Enrichment> 8) by the 105 common genes of L3 and L4. They are “hsa04978: Mineral absorption”, “hsa04974: Protein digestion and absorption” and “hsa04512: ECM-receptor interaction”. A main difference between L3 and L4 is that the former, but not the latter, is enriched (p < 0.001 and Fold Enrichment> 2) with the genes involved in “hsa04151:PI3K-Akt signaling pathway”.

**Figure 6:**
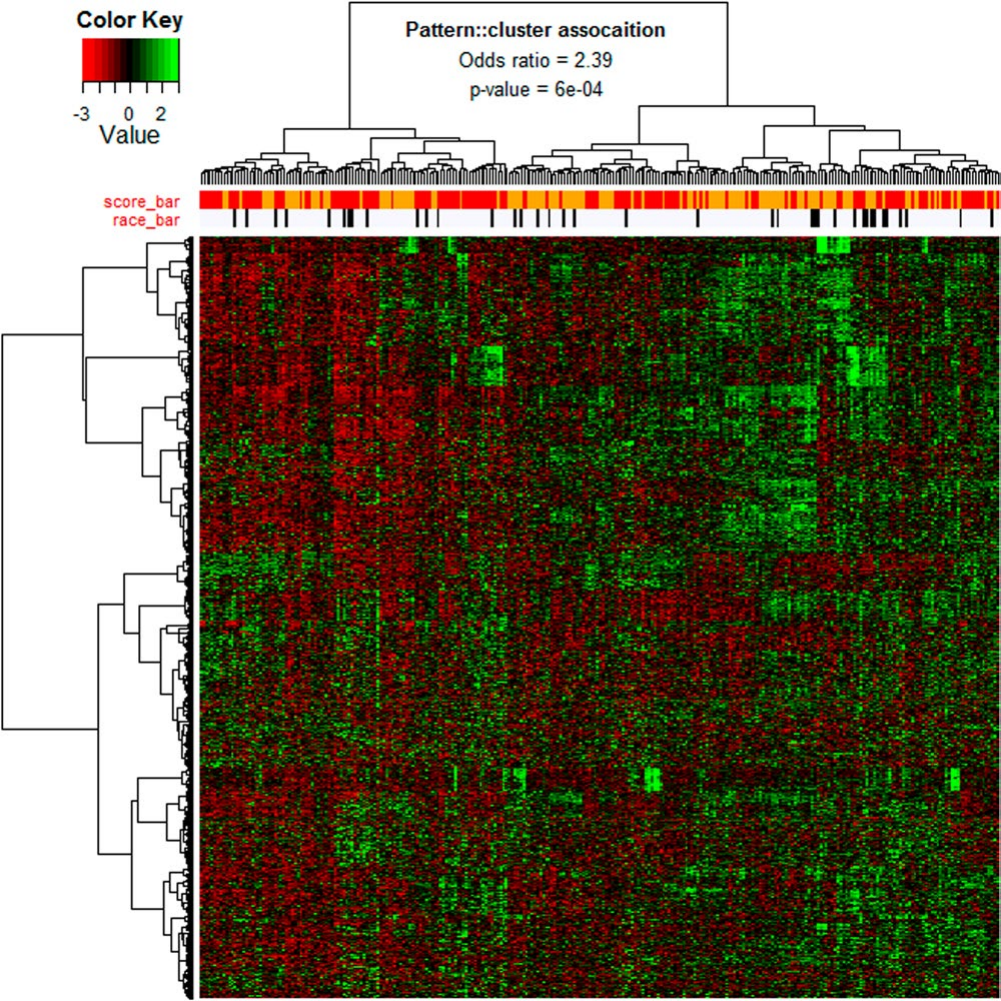
Clustering and heatmap analysis of the digital gene expression profiling of TCGA samples (i.e. dataset Ex-1). 1000 genes with top variability coefficients in the expression levels across tumor samples are focused. The rows represent genes and the columns represent tumors. The “score-bar” indicates the Gleason patterns of tumor specimens with red being GP-4 and orange being GP-3. The “race-bar” indicates the races of patients with black being AAs and white being EAs. The printed Odds ratio and p-value are for the associations between the tumor clusters (k = 2) and Gleason pattern categories (GP-3 and GP-4). The associations between the tumor clusters and patient races are not significant. As such, those statistics are not reported.

Apparently, the genes with different expression levels between AA and EA specimens of the same Gleason pattern are rare, and the contribution of the Gleason patterns to the variability of gene expression activity is much more significant than patient races. This conclusion is verified by the same comparison analysis performed on the composite microarray gene expression data (Supplementary Fig. 4A–D). It is also supported by the results of clustering analysis (Fig. 6, Supplementary Fig. 5), which show that the tumor clusters established on the expression profiling of 1000 genes with top expression variability are associated with Gleason patterns but not patient races.

## Discussion

The primary conclusion of this study is that, prostate tumors being more lethal in AAs than in EAs is reasonable regarding AAs’ higher PHG statistic, while high grade alone could not imply aggressiveness. However, this notion is questionable when the comparison is concentrated on tumor samples within the same grade category. The following are the supporting observations and evidence for this conclusion. First, based on the records of 48 representative registries (i.e. counties and parishes), the nation-wide PHG in AAs is ~1.2 times of that in EAs; Second, for the patients with tumors in the same grade category (HG or LG), the survival stratification between races is not significant in most counties (or parishes) located in different states; and third, the genes with different expression levels between AA and EA patients with cancers in the same grade category are very limited in number. These results, especially the second one, hold a remarkable implication for erasing racial disparities in prostate cancer. That is, “Equal grade, equal outcomes” is not only a verifiable hypothesis but also an achievable public health goal. In some areas, such as DeKalb County in Georgia and Jefferson Parish in Louisiana, AA and EA patients have the similar outcomes. The experiences of these regions in patient surveillance and therapy could be useful to some other areas, such as Fulton County in Georgia and Wayne County in Michigan, where the mortality disparity is substantial. The reason is that, while the between-race survival differences of same-grade patients observed in the latter counties or parishes reflect the findings of several publications^28,29^, they may purely be an artifact of imbalances in treatment.

Our opinion about the achievability of the “Equal grade, equal outcomes” goal is largely supported by the results of a recent publication^30^, which suggests that racial inequality in prostate cancer outcome is mainly due to socioeconomic imbalances rather than biological factors. Based on a comprehensive analysis of the data of three cohorts, i.e. SEER, a pool of four randomized clinical trials and an equal access health care system, Dess *et al*. found that prostate cancer–specific mortality by race did not appear to differ in the equal-access health care system, and the outcomes were even better for black than for white patients in well-designed and well-conducted clinical trials. Regarding the analysis performed on the SEER data, Dess *et al*. used a propensity model to adjust the statistics of patient outcomes for socioeconomic imbalances and clinical factors such as biopsy Gleason score (corresponding to “grade” in our study). As a result, they did not identify substantial racial disparity in prostate cancer specific mortality.

On the other hand, our study and results are subject to the limitations of using the SEER data and some conclusions could be weakened. Especially, the causes of ~20% of death cases are missing in the data but the relevance of prostate cancers in those deaths cannot be excluded. This could lead to an underestimate of PSMR and temper the benefit of a PSMR-based survival analysis. Moreover, SEER does not have a mechanism to guarantee the consistence of the protocols in measuring the Gleason scores (GS) of tumors across hospitals and, therefore, miscoding for GS-based grades of tumors is unavoidable. This potentially compromises the accuracy of the disparity evaluation performed in this study.

There are other points relevant to the aforementioned conclusion worthy of further discussion. *The first* is the biological functions of the genes that show “significant” differences between the AAs’ and EAs’ tumors. Using the expression dataset Ex-1 (or Ex-2), we identify 49 and 51 (or 50 and 74) differentially-expressed genes in the contrasts, AA&GP-3 versus EA&GP-3 and AA&GP-4 versus EA&GP-4. The functional enrichment analysis of those genes does not show any relevance to tumor mortality. Those genes also hardly overlap with the 46 significant genes identified in a previous study on racial disparity of gene expression^10^, which are enriched with a few gene ontology terms related to immune response. *The second* is whether there is a disparity in the mutation spectrum of tumor cells. Because of the lack of a predominant cancer gene which is widely mutated over prostate tumors^31^ and the limited sample size of AA patients, our preliminary analysis of the TCGA somatic mutation data does not identify any other genes with significantly different mutation frequencies between AAs and EAs. Although ETS fusions, predominated by TMPRSS2-ERG fusions, are two-times popular in EAs’ tumors compared to AAs’ tumors^32–35^, their effect on patient outcomes is controversial^36–38^. *The third* is the possibility of attributing the observed disparity in mortality rate to the potential disparity in cancer progression. While, to our knowledge, this point has been barely addressed by previous studies, the possibility is small in our opinion. The reason is that, in general, a GP-3 tumor cannot directly progress into a GP-4 tumor^16,39^.

Due to the complex demographical and socio-economic factors as well as the measurement errors arising from the limited sizes of AA patients in some registries, it is no wonder that there is a substantial variability in R-PHGs and R–TMRs, i.e. registry-specific PHG and TMR measures. However, a positive correlation between the R-PHGs and T–TMRs is expected if there is no demographical disparity in patient surveillance and therapy. Here, we do observe such a relationship in EAs but not in AAs. This represents a unique inequality. The underlying reason may be that, among AA communities, there is substantial variability in access to effective surveillance and therapy that masks the effect of cancer grade on patient survival. Therefore, this disparity could be attributed to AAs’ poor socio-economic situation in some communities.

It is well known that PCa patients with tumors diagnosed before the age of 50 years are rare and that such early-onset patients are more frequent in AAs than in EAs. According to the records of the working dataset SEER-WD, 2030 EA men and 1105 AA men are within this age category (i.e. <50 years). They account for 3.0% and 6.1% of the EA patients and AA patients, respectively. Importantly, we find a relatively lower PHG in those early-onset AA patients whose death is attributable to prostate tumors. As shown in Fig. 3C, while the PHG quantity of those AA patients is up to 83% but this level is still lower than that of the patients in other age segments. This pattern sharply contrasts with that observed in EAs, where nearly all of the early-onset patients who died of prostate cancer have the high-grade tumors. The EA’s pattern would be expected because a low-grade prostate tumor should not be lethal to a younger man whose immune system is strong, in general. The AA’s pattern holds some epidemiological and genetic implications. First, some of the younger AA men with low-grade prostate cancers may not receive appropriate surveillance. Second, the low-grade prostate cancers observed in AA men younger than 45 years may represent a unique cancer subtype with inheritable risk factors. Such a hypothesis is originated from the fact that hereditary cancer syndromes are associated with an earlier age of onset compared to sporadic cancers^40–42^.

## Supplementary information

Supplementary Figure S1-S5.

## Data Availability

The used TCGA and GEO data reside at https://gdc-portal.nci.nih.gov/legacy-archive/search/f and https://www.ncbi.nlm.nih.gov/geo/, respectively.

## References

[1] Siegel R, Ma J, Zou Z, Jemal A (2014). Cancer statistics, 2014. CA Cancer J. Clin..

[2] NCI. Prostate Cancer Treatment (PDQ®)–Health Professional Version, <https://www.cancer.gov/types/prostate/hp/prostate-treatment-pdq#cit/section_1.21> (2018).

[3] Noone, A. M. *et al*. SEER Cancer Statistics Review, 1975–2015, National Cancer Institute. Bethesda, MD, https://seer.cancer.gov/csr/1975_2015/ (2018).

[4] Haiman CA (2011). Characterizing genetic risk at known prostate cancer susceptibility loci in African Americans. Plos Genet..

[5] Chang BL (2005). Genome-wide screen for prostate cancer susceptibility genes in men with clinically significant disease. Prostate.

[6] Wu I, Modlin CS (2012). Disparities in prostate cancer in African American men: what primary care physicians can do. Cleve Clin. J. Med..

[7] Powell IJ, Bock CH, Ruterbusch JJ, Sakr W (2010). Evidence supports a faster growth rate and/or earlier transformation to clinically significant prostate cancer in black than in white American men, and influences racial progression and mortality disparity. J. Urol..

[8] Kim HS (2011). Prostate biopsies from black men express higher levels of aggressive disease biomarkers than prostate biopsies from white men. Prostate Cancer Prostatic Dis..

[9] Petrovics G (2015). A novel genomic alteration of LSAMP associates with aggressive prostate cancer in African American men. EBioMedicine.

[10] Wallace TA (2008). Tumor immunobiological differences in prostate cancer between African-American and European-American men. Cancer Res..

[11] Timofeeva OA (2009). Enhanced expression of SOS1 is detected in prostate cancer epithelial cells from African-American men. Int. J. Oncol..

[12] Fowler JE, Terrell F (1996). Survival in blacks and whites after treatment for localized prostate cancer. J. Urol..

[13] Tewari A (2005). Racial differences in serum prostate-specific antigen (PSA) doubling time, histopathological variables and long-term PSA recurrence between African-American and white American men undergoing radical prostatectomy for clinically localized prostate cancer. BJU Int..

[14] Bozeman C, Williams BJ, Whatley T, Crow A, Eastham J (2000). Clinical and biopsy specimen features in black and white men with clinically localized prostate cancer. South. Med. J..

[15] SEER. SEER Program Coding and Staging Manual, https://seer.cancer.gov/archive/manuals/2012/AppendixC/prostate/coding_guidelines.pdf (2012).

[16] Lavery HJ, Droller MJ (2012). Do Gleason patterns 3 and 4 prostate cancer represent separate disease states?. J. Urol..

[17] Stamey TA, McNeal JE, Yemoto CM, Sigal BM, Johnstone IM (1999). Biological determinants of cancer progression in men with prostate cancer. JAMA.

[18] Cheng L, Davidson DD, Lin H, Koch MO (2007). Percentage of Gleason pattern 4 and 5 predicts survival after radical prostatectomy. Cancer.

[19] Cancer Genome Atlas Research N (2015). The Molecular Taxonomy of Primary Prostate Cancer. Cell.

[20] Li B, Dewey CN (2011). RSEM: accurate transcript quantification from RNA-Seq data with or without a reference genome. BMC Bioinforma..

[21] Li B, Ruotti V, Stewart RM, Thomson JA, Dewey CN (2010). RNA-Seq gene expression estimation with read mapping uncertainty. Bioinformatics.

[22] Taylor BS (2010). Integrative genomic profiling of human prostate cancer. Cancer Cell.

[23] Klein EA (2015). A genomic classifier improves prediction of metastatic disease within 5 years after surgery in node-negative high-risk prostate cancer patients managed by radical prostatectomy without adjuvant therapy. Eur. Urol..

[24] Zhao SG (2016). The Landscape of Prognostic Outlier Genes in High-Risk Prostate Cancer. Clin. Cancer Res..

[25] McCall MN, Jaffee HA, Irizarry RA (2012). fRMA ST: frozen robust multiarray analysis for Affymetrix Exon and Gene ST arrays. Bioinformatics.

[26] Zhang, W., Flemington, E. K. & Zhang, K. Gene expression analysis reveals a pitfall in the molecular research of prostate tumors relevant to Gleason scores (submitted). (2019). 10.1142/S0219720020500328 PMC8627177 32938283

[27] Huang da W, Sherman BT, Lempicki RA (2009). Systematic and integrative analysis of large gene lists using DAVID bioinformatics resources. Nat. Protoc..

[28] Mullins CD, Onukwugha E, Bikov K, Seal B, Hussain A (2010). Health disparities in staging of SEER-medicare prostate cancer patients in the United States. Urology.

[29] Steele CB, Li J, Huang B, Weir HK (2017). Prostate cancer survival in the United States by race and stage (2001–2009): Findings from the CONCORD-2 study. Cancer.

[30] Dess RT (2019). Association of Black Race With Prostate Cancer-Specific and Other-Cause Mortality. JAMA Oncol..

[31] Bunz, F. Principles of cancer genetics. (Springer, 2008).

[32] Khani F (2014). Evidence for molecular differences in prostate cancer between African American and Caucasian men. Clin. Cancer Res..

[33] Magi-Galluzzi C (2011). TMPRSS2-ERG gene fusion prevalence and class are significantly different in prostate cancer of Caucasian, African-American and Japanese patients. Prostate.

[34] Rosen P (2012). Differences in frequency of ERG oncoprotein expression between index tumors of Caucasian and African American patients with prostate cancer. Urology.

[35] Zhou CK (2017). TMPRSS2:ERG Gene Fusions in Prostate Cancer of West African Men and a Meta-Analysis of Racial Differences. Am. J. Epidemiol..

[36] John J, Powell K, Conley-Lacomb MK, Chinni SR (2012). TMPRSS2-ERG Fusion Gene Expression in Prostate Tumor Cells and Its Clinical and Biological Significance in Prostate Cancer Progression. J. Cancer Sci. Ther..

[37] Dal Pra A (2013). TMPRSS2-ERG status is not prognostic following prostate cancer radiotherapy: implications for fusion status and DSB repair. Clin. Cancer Res..

[38] Gasi Tandefelt D, Boormans J, Hermans K, Trapman J (2014). ETS fusion genes in prostate cancer. Endocr. Relat. Cancer.

[39] Sowalsky AG (2017). Gleason Score 7 Prostate Cancers Emerge through Branched Evolution of Clonal Gleason Pattern 3 and 4. Clin. Cancer Res..

[40] Garber JE, Offit K (2005). Hereditary cancer predisposition syndromes. J. Clin. Oncol..

[41] Brandt A, Bermejo JL, Sundquist J, Hemminki K (2008). Age of onset in familial cancer. Ann. Oncol..

[42] Knudson AG (1971). Mutation and cancer: statistical study of retinoblastoma. Proc. Natl Acad. Sci. USA.

