# Supplementary material for: SEER and Gene Expression Data Analysis Deciphers Racial Disparity Patterns in Prostate Cancer Mortality and the Public Health Implication
Source: Sci Rep. 2020 Apr 22;10:6820. doi: 10.1038/s41598-020-63764-4 (PMC7176737; doi:10.1038/s41598-020-63764-4)
Supplement: Supplementary file 1 — Supplementary Figure S1-S5. [file 41598_2020_63764_MOESM1_ESM.pdf]

*Supplementary files of*

# "SEER and Gene Expression Data Analysis Deciphers Racial Disparity Patterns in Prostate Cancer Mortality and the Public Health Implication"

Wensheng Zhang[1], Yan Dong[2], Oliver Sartor[3], Erik K. Flemington[4] and Kun Zhang[1]§

[1]Bioinformatics Core of Xavier NIH RCMI Center of Cancer Research; Department of Computer Science, Xavier University of Louisiana, New Orleans, LA, 70125, USA;
[2]Department of Structural and Cellular Biology, Tulane University School of Medicine, Tulane Cancer Center, New Orleans, LA, 70112, USA;
[3]Department of Medicine, Tulane University School of Medicine, Tulane Cancer Center, New Orleans, LA, 70112, USA;
[4]Department of Pathology, Tulane University School of Medicine, Tulane Cancer Center, New Orleans, LA, 70112, USA;

§ Corresponding author: kzhang@xula.edu

## Content:

Supplementary Figure S1, page 2

Supplementary Figure S2A, B, page 2

Supplementary Figure S3, page 3

Supplementary Figure S4A-D, page 4

Supplementary Figure S5, page 5

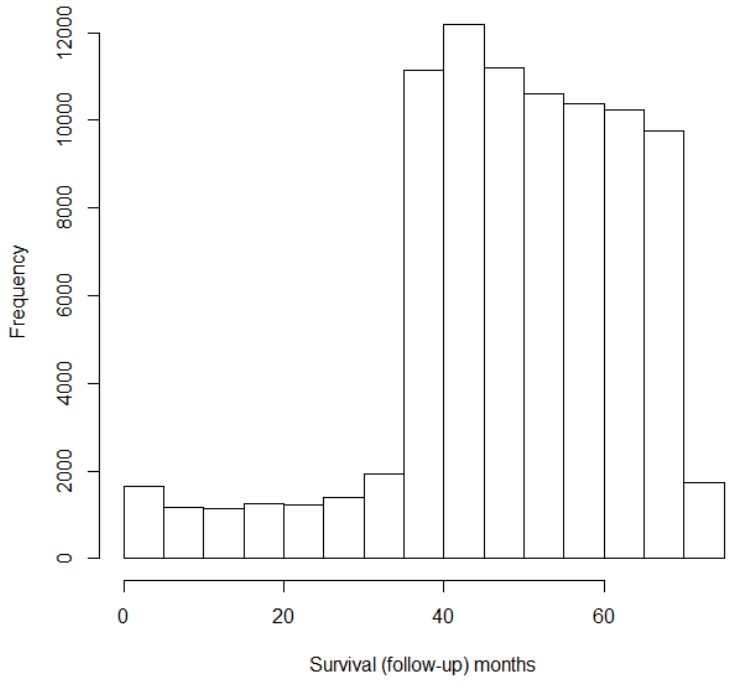


**Supplementary Figure S1. Distribution of survival (or follow-up) time of the patients in the working dataset SEER-WD.**

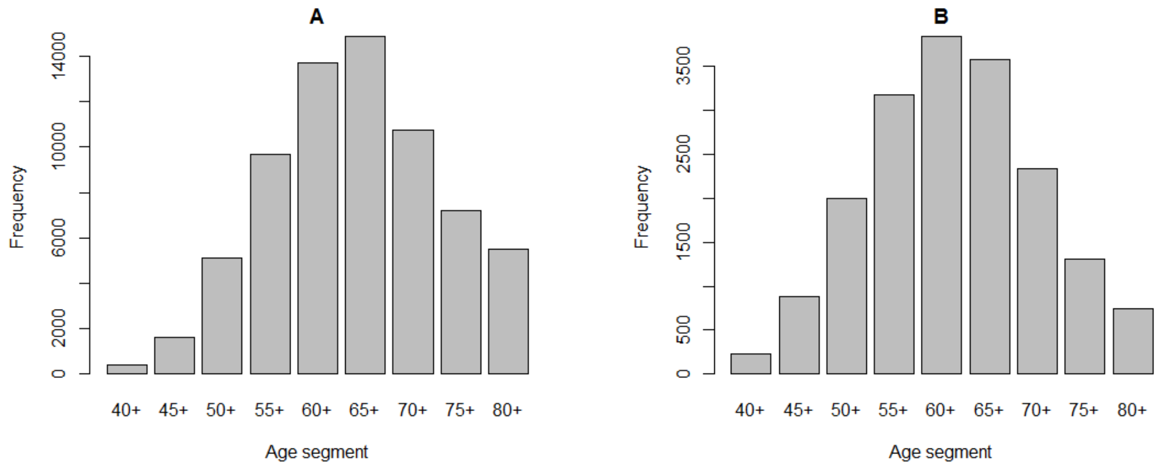


**Supplementary Figure S2A, B. Distributions of ages at the initial diagnosis dates of the EA patients (A) and AA patients (B) in the working dataset SEER-WD.**

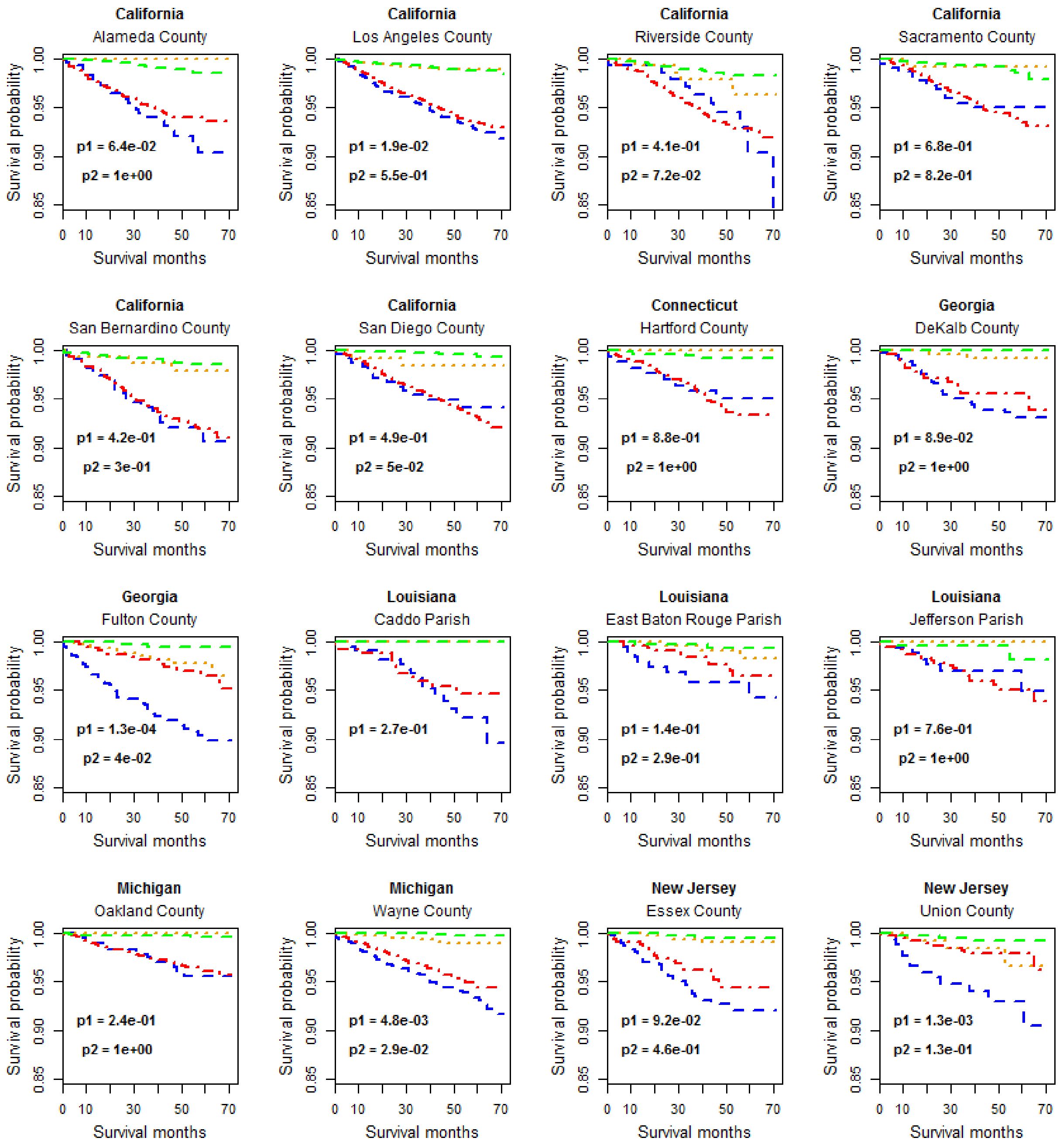


**Supplementary Figure S3. Survival analyses of the patients in 16 representative registries (i.e. counties or parishes) regarding prostate cancer specific mortality.** Green: EA-LG, i.e. EA patients with low-grade cancers. Red: EA-HG, i.e. EA patients with high-grade cancers. Orange: AA-LG, i.e. AA patients with low-grade cancers. Blue: AA-HG, i.e. AA patients with high-grade cancers. p1: p-value for the comparison between EA-HG and AA-HG. p2: p-value for the comparison between EA-LG and AA-LG.

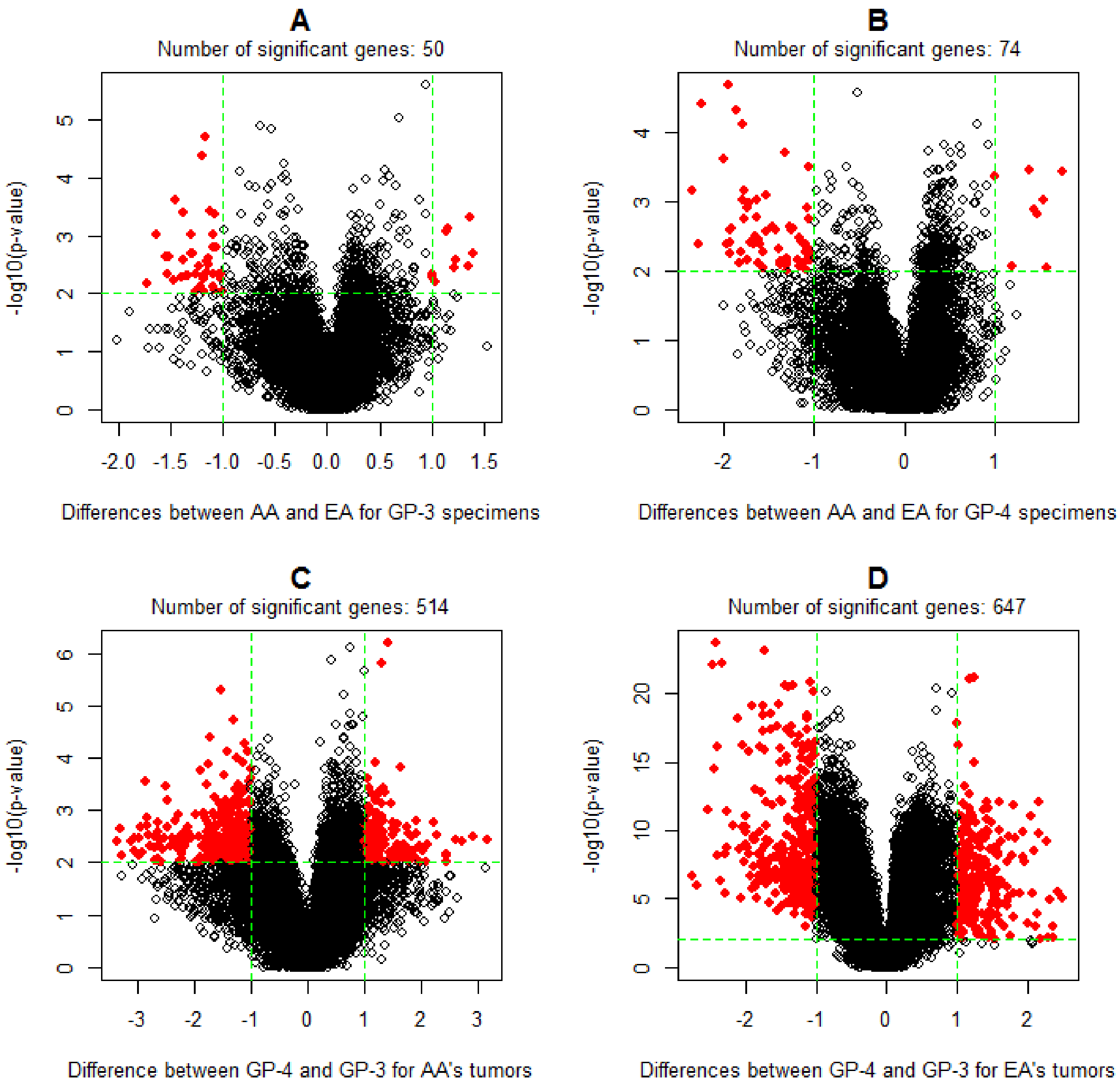


**Supplementary Figure S4A-D. Identification of differentially expressed genes between specimen groups defined by patient races and Gleason patterns.** The dataset Ex-2, i.e. the composite microarray gene expression data of multi-cohorts samples, is used. Significant genes are indicated by red points. The analysis is based on the log2 transformed gene expression levels. Thus, the cutoffs (1 and -1) correspond to a 2-fold change.

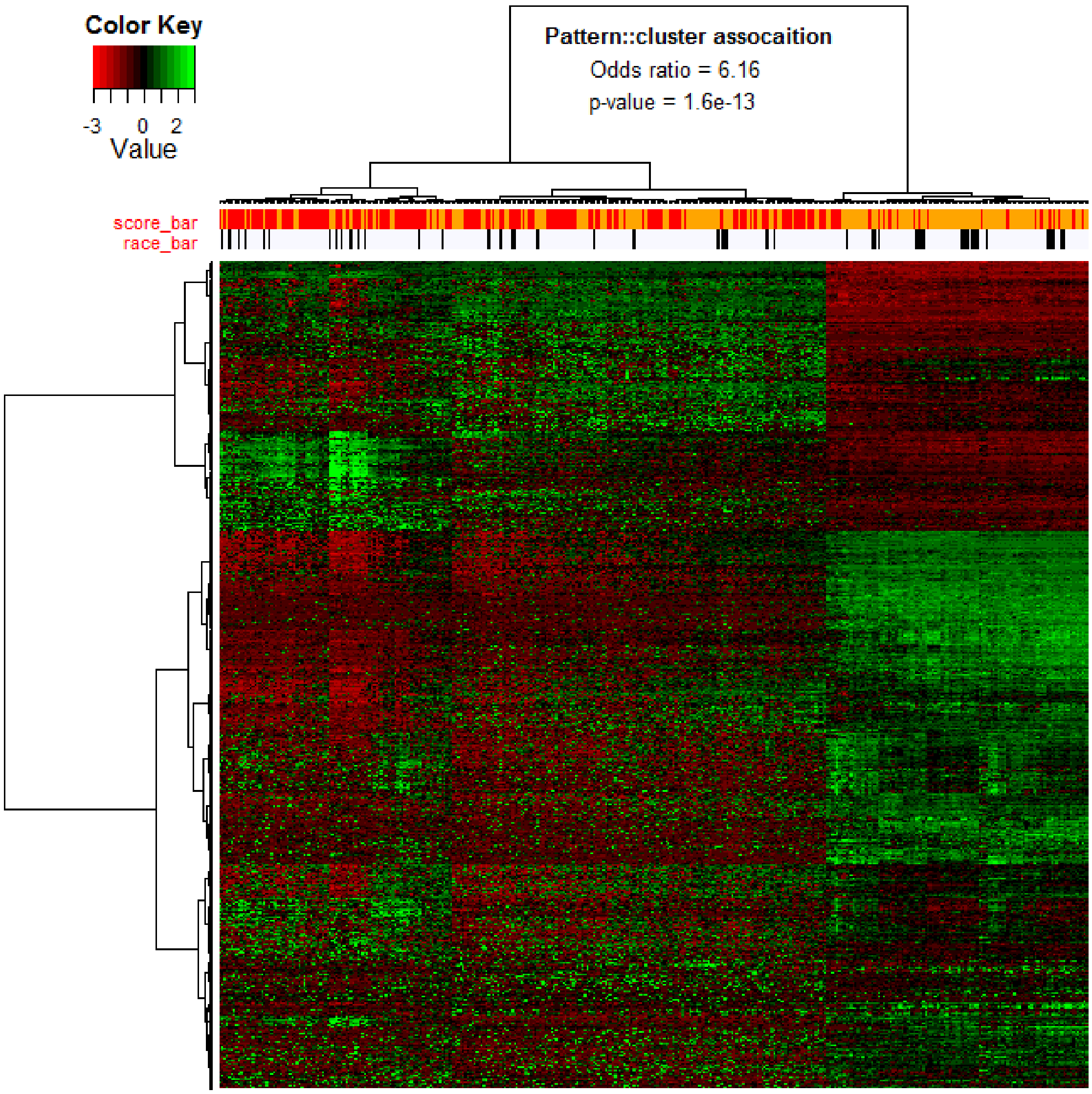


**Supplementary Figure S5. Clustering and heatmap analysis of the composite microarray gene expression profiling (Ex-2) of multi-cohorts samples.** 1000 genes with top variability coefficients in the expression levels across tumor samples are focused. The rows represent genes and the columns represent tumors. The "score-bar" indicates the Gleason patterns of tumor specimens with red being GP-4 and orange being GP-3. The "race-bar" indicates the races of patients with black being AAs and white being EAs. The printed Odds ratio and p-value are for the associations between the tumor clusters (k=2) and Gleason pattern categories (GP-3 and GP-4). The associations between the tumor clusters and patient races are not significant. As such, those statistics are not reported.
